# The Bi-Directional Prediction of Carbon Fiber Production Using a Combination of Improved Particle Swarm Optimization and Support Vector Machine

**DOI:** 10.3390/ma8010117

**Published:** 2014-12-30

**Authors:** Chuncai Xiao, Kuangrong Hao, Yongsheng Ding

**Affiliations:** 1 College of Information Sciences and Technology, Donghua University, Shanghai 201620, China; E-Mails: 1119110@mail.dhu.edu.cn (C.X.); ysding@dhu.edu.cn (Y.D.); 2 Engineering Research Center of Digitized Textile & Apparel Technology, Ministry of Education, Shanghai 201620, China

**Keywords:** support vector machine, particle swarm optimization, intelligent computing, cell communication mechanism, carbon fiber, bi-directional prediction

## Abstract

This paper creates a bi-directional prediction model to predict the performance of carbon fiber and the productive parameters based on a support vector machine (SVM) and improved particle swarm optimization (IPSO) algorithm (SVM-IPSO). In the SVM, it is crucial to select the parameters that have an important impact on the performance of prediction. The IPSO is proposed to optimize them, and then the SVM-IPSO model is applied to the bi-directional prediction of carbon fiber production. The predictive accuracy of SVM is mainly dependent on its parameters, and IPSO is thus exploited to seek the optimal parameters for SVM in order to improve its prediction capability. Inspired by a cell communication mechanism, we propose IPSO by incorporating information of the global best solution into the search strategy to improve exploitation, and we employ IPSO to establish the bi-directional prediction model: in the direction of the forward prediction, we consider productive parameters as input and property indexes as output; in the direction of the backward prediction, we consider property indexes as input and productive parameters as output, and in this case, the model becomes a scheme design for novel style carbon fibers. The results from a set of the experimental data show that the proposed model can outperform the radial basis function neural network (RNN), the basic particle swarm optimization (PSO) method and the hybrid approach of genetic algorithm and improved particle swarm optimization (GA-IPSO) method in most of the experiments. In other words, simulation results demonstrate the effectiveness and advantages of the SVM-IPSO model in dealing with the problem of forecasting.

## 1. Introduction

Carbon fibers are produced mainly from polyacrylonitrile (PAN), rayon, and pitch. Attributing to inherent structural composites, the PAN-based carbon fibers have maintained their predominance as engineering materials up to the present. It has been documented that 90% of the world’s total carbon fibers used today are made from the PAN precursor [1]. Their high specific modulus and outstanding fatigue characteristics, combined with their stiffness and lower weight, make these fibers attractive for mass applications ranging from sporting goods to engineering components [2].

Producing a high quality carbon fiber is not an easy thing, since the technological process for carbon fiber is a complex nonlinear system which involves many process parameters that must be carefully optimized and adjusted. Most of the previous work was focused on analyzing the properties of carbon fibers by means of physical or chemical instruments. Liu *et al.* [3] referred to a surface treatment technique of electrochemical oxidation to improve interfacial bonding strength and tensile strength of carbon fibers. Wang *et al.* [4] investigated the chemical element potassium permanganate modification for carbon fibers during the heat treatment process by differential scanning calorimetry, infrared spectra, elemental analysis, and X-ray photoelectron spectroscopy. Rahman *et al.* [5] referred to the residence time of 3 s as being the most suitable residence time for producing carbon fibers in a solvent-free coagulation process. Experimental data suggest that the Young’s modulus of carbon fibers can reach a highest value of 2.55 Gpa. Liang *et al.* [6] used a bio-inspired intelligent cooperative controller to provide a plan for a stretching process for fiber production. Rennhofer *et al.* [7] investigated the structural change of carbon fibers with the use of an X-ray testing device at high temperatures under load. Belyaev *et al.* [8] investigated the kinetics of carbon fibers in oxidative stabilization by differential scanning calorimetry data. Chen *et al.* [9] proposed a hybrid model of genetic algorithm and improved particle swarm optimization to optimize the radial basis function neural network for real-time predicting of the carbon fiber manufacturing process. According to all the kinds of descriptions mentioned above, we know that they mostly previously analyzed properties with the aid of different instruments [10], considering solely relationship between the productive parameters and the fiber properties in the literature. This situation resulted for two main reasons, on the one hand, numerous researchers in materials science had different perspectives in the study of the productive process, while on the other hand, the technological process for carbon fiber is a nonlinear system, containing a lot of separate processes: polymerization, spinneret, coagulating baths, washing, stretching, applying oil, drying, pre-oxidation, carbonization, and more. These process can be regarded as subsystems, each subsystem has its own control parameters. These parameters affect and restrict the performance of the whole system directly, whereas they are not only affected by interrelation and coupling among subsystems but also by the external environment. Therefore, it is difficult to establish a precise mathematical model to represent linearly the relation between properties indices and productive parameters.

With the rise of intelligent algorithms, the theory of intelligent algorithm has provided a powerful tool of systematic research for analyzing the unknown complex nonlinear system. Among them, artificial neural network (ANN) has proved to be an excellent adaptive method with dark-box operating performance, powerful study and generalized ability to deal with modeling the dynamic process for process control. Kadi [11] used the ANN to predict mechanical modeling for fiber-reinforced composite materials. Yu *et al.* [12] used a fuzzy ANN to predict the fabric hand in different fabric specimens. However, ANN needs a long training time because its topology is not compact enough. Then RNN was adopted to compensate for the weaknesses of ANN. Du *et al.* [13] investigated the center selection of multi-output RNN. Roy *et al.* [14] investigated the learning theory of the RNN. Hong *et al.* [15] presented a novel topology of the RNN, referred to as the boundary value constraints. Huang *et al.* [16] investigated the function approximation of the RNN. Qiao *et al.* [17] presented a self-organizing RNN to identify nonlinear systems and the model. Wang *et al.* [18] proposed a forecasting method based on self-correcting RNN for adapting changing conditions. However, the training procedure for ANN models is not only time consuming but it is also possible to get trapped in local minima and subjectively in selecting the model architecture [19]. SVM is a relatively new artificial intelligence technique which is increasingly being applied to geotechnical problems and has yielded encouraging results [20,21]. SVM implements the structural risk minimization principle rather than the empirical risk minimization principle implemented by most traditional ANN models [22,23]. Based on this principle, SVM achieves an optimum network structure; meanwhile, it can lower the global error of the model. It raises the generalization capability of the model, which is more prominent in small-sample learning. SVM have found a wide application in the fields of pattern recognition, bio-informatics, and nonlinear regression estimation problems. Particularly, support vector regression (SVR) is an extension of SVM. Wang *et al.* [24] proposed a hybrid load forecasting model combining differential evolution algorithm and SVR. Gilan *et al.* [25] developed a hybrid SVR and PSO model to predict the compressive and rapid chloride penetration test (RCPT) results of concretes containing metakaolin. Hong [26] developed a hybrid SVR and chaotic particle swarm optimization model to predict electric load. Kang *et al.* [27] developed swarm intelligence approaches to optimize power flow problems in power networks. Liang *et al.* [28] developed an adaptive PSO method based on clustering to solve multimodal function optimization problems.

In this paper, we propose the SVM-IPSO hybrid model to bi-directionally forecast a productive process for carbon fiber. We use IPSO to tune the parameters in SVM. With this model, we can predict the performance of carbon fiber and obtain a design scheme for new carbon fiber.

The main contributions of this article include the following: (1) we propose IPSO, which pushes forward the development of intelligent computing; (2) the hybrid SVM-IPSO model is used to set up a bi-directional prediction approach for carbon fiber; (3) with the proposed model, we can online forecast the productive parameters and the properties of carbon fiber, adjust technical parameters in time, reduce not only wasted time but also energy consumption.

The main structure of this article is organized as follows: The first section describes the research motivation of this paper. The second section introduces the regression algorithm of SVM. The third section introduces the parameters for selecting SVM based on IPSO. The fourth section testifies the performance of the proposed model with the experimental data and compares the results with other methods. The fifth section discusses the research conclusion.

## 2. Carbon Fiber Production and Its Bi-Directional Optimization

### 2.1. The Process of Carbon Fiber Production

**Figure 1:**
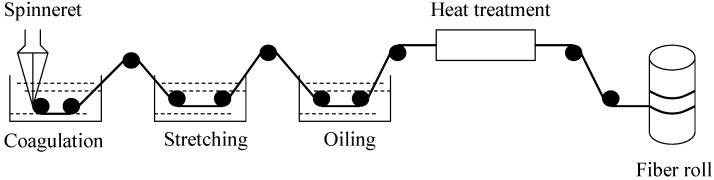
The technological process of carbon fiber production.

The technological process for carbon fiber is a sufficiently complex system. It is a very meaningful research subject to control the performance of the carbon fiber process in as small a range as we possibly can. For this purpose, we should observe the parameters of the productive process in real-time, synchronously predict the product properties of the carbon fiber, after which we can determine the defective carbon fiber, timely adjust the parameters of the productive process, avoid reducing the quality of the carbon fiber, and prevent significantly wasting of raw materials. The production of carbon fiber mainly comprises several processes, such as coagulation, stretching, oiling, and heat treatment. Figure 1 shows the main technological process of carbon fiber production.

### 2.2. The Bi-Directional Prediction Methods for Carbon Fiber Production

The bi-directional prediction methods include forward prediction and backward prediction. Forward prediction is the process of predicting property indexes from productive parameters. It means that when the productive parameters of carbon fiber are changed, the corresponding performance of carbon fiber will fluctuate accordingly. In other words, if the productive parameters have little differences, there must be the same among the carbon fiber performances. The backward prediction is a reverse process of the forward prediction, so the process can be seen as a method for designing new types of carbon fiber. In the early stage, we design a new kind of carbon fiber through previous experience, then we test this kind of carbon fiber with chemical and physical instruments to verify its expected performance, get inconsistent results, adjust the corresponding parameters in a new scheme, produce carbon fiber products again, repeat this process of production, testing, and adjusting parameters until we get a satisfactory performance of the carbon fiber. From the foregoing, this method wastes a lot of raw materials, energy, and time. The worst thing is that, if we choose inappropriate parameters for the manufacturing equipment, it will cause damage to the corresponding equipment. For this reason, a better way to solve this problem is to use computer simulation in the whole productive process, avoid unilateral decisions made by workers with interests and experience, and give out more reasonable control parameters based on the actual production process.

**Figure 2:**
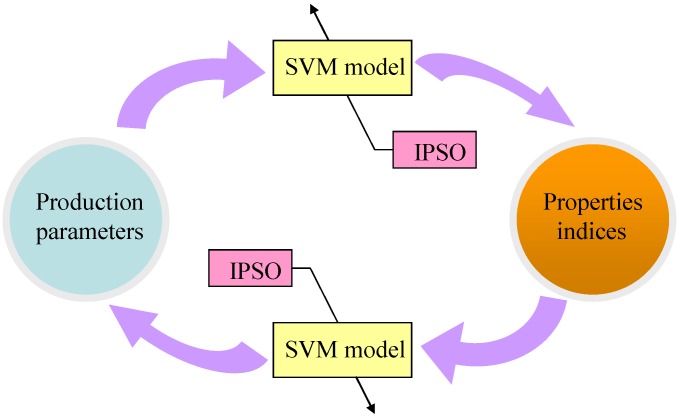
Bidirectional prediction model of carbon fiber production.

In this paper, we propose a bi-directional prediction model of carbon fiber production based on SVM. We adopt IPSO to tune the parameters of the model. The overall chart of bi-directional prediction approaches are as shown in Figure 2.

## 3. Methodology of the SVM-IPSO Model

### 3.1. The SVM Model

The SVM, proposed by Vapnik in 1995 [29], is widely used for many machine learning problems such as bio-informatics, pattern recognition, linear regression analysis and nonlinear regression estimation problems.

The basic principle is as follows [30]: given a training data ${\left\{x_{i},y_{i}\right\}}_{i=1}^{N}$, where $x_{i}\in R^{n}$ is the input vector and $y_{i}\in R$ is the corresponding output of input *x_i_*, and *N* is the total number of the input vectors. By nonlinear mapping φ(*x*), the data are mapped nonlinearly from the original feature space to a high-dimensional feature space, thus, it can be approximated in a linear way as follows:

(1) $$f(x)=w^{T}\text{φ}(x)+b$$

where *f*(*x*) denotes the regression estimate function; *w* denotes the weight vector; and *b* denotes the bias vector.

As mentioned above, the problem of regression approximation is equivalent to minimizing the empirical risk,

(2) $$R(f)=\frac{1}{2}{\left\|w\right\|}^{2}+\frac{C}{N}\sum_{i=1}^{N}{\text{Θ}}_{\xi }(y_{i},f(x))$$

where *C* and ξ are regularized constants; Θ*_ξ_*(*y_i_*, *f*(*x*)) denotes the ξ-insensitive loss function and is defined as Equation (3)

(3) $${\text{Θ}}_{\xi }(y_{i},f(x))=\{\begin{array}{l} \left|f(x)-y_{i}\right|-\text{ξ},\begin{array}{c} \begin{array}{cc} if & \left|f(x)-y_{i}\right| \end{array} \end{array}\geq \text{ξ}\\ 0,\begin{array}{c} \begin{array}{c} \begin{array}{c} otherwise \end{array} \end{array} \end{array} \end{array}$$

Then, we introduce two positive slack variables ${\text{ε}}_{i}^{´}$ and ε*_i_* to simplify the risk function *R*(*f*) in Equation (2). Thus, Equation (2) is transformed into the objective function shown as follows:

(4) $$Min_{w,b,\text{ε}´,\text{ε}}R=\frac{1}{2}{\left\|w\right\|}^{2}+C\sum_{i=1}^{N}\left({\text{ε}}_{i}^{´}+{\text{ε}}_{i}\right)$$

with the constraints

(5) $$\{\begin{array}{l} y_{i}-w^{T}\text{φ}(x_{i})-b\leq \text{ξ}+{\text{ε}}_{i}^{´}\begin{array}{cc} , & i=1,2,\cdots ,N \end{array}\\ -y_{i}+w^{T}\text{φ}(x_{i})+b\leq \text{ξ}+{\text{ε}}_{i}\begin{array}{cc} , & i=1,2,\cdots ,N \end{array}\\ {\text{ε}}_{i}^{´}\geq 0\begin{array}{cc} \begin{array}{c} , \end{array} & i=1,2,\cdots ,N \end{array}\\ {\text{ε}}_{i}\geq 0\begin{array}{cc} \begin{array}{c} , \end{array} & i=1,2,\cdots ,N \end{array} \end{array}$$

where ε*i* is the lower training error (${\text{ε}}_{i}^{´}$ is the upper) subjected to the ξ-insensitive loss function.

After the convex quadratic programming problem with constraints is solved, the weight vector *w* in Equation (1) is obtained as Equation (6),

(6) $$w=\sum_{i=1}^{N}\left({\text{β}}_{i}^{´}-{\text{β}}_{i}\right)\text{φ}(x_{i})$$

where ${\text{β}}_{i}^{´}$, ${\text{β}}_{i}$ are the Lagrangian multipliers and are obtained by solving a convex quadratic optimization.

Finally, the regression function is obtained as the following equation:

(7) $$f(x)=\sum_{i=1}^{N}\left({\text{β}}_{i}^{´}-{\text{β}}_{i}\right)K(x_{i},x_{j})+b$$

where *K*(*x_i_*, *x_j_*) is called the kernel function, which is the inner product of φ(*x_i_*) and φ(*x_j_*), that is, *K*(*x_i_*, *x_j_*)= φ(*x_i_*)•φ(*x_j_*). The values of φ(*x_i_*) and φ(*x_j_*) are produced by mapping *x_i_* and *x_j_* into the higher-dimensional feature space, respectively. It can be shown that any function meeting Mercer’s criteria [29] can be used as the kernel function.

However in general, three different types of kernel functions are commonly used, e.g., the sigmoid kernel function, the polynomial kernel function, and the Gaussian RBF kernel function. The Gaussian RBF is a most used for the kernel function because of the fewer free variables that need to be set and an excellent nonlinear forecasting performance [31]. Therefore, in this study, the Gaussian RBF was selected (as shown in Equation (8)) as the kernel function:

(8) $$K(x_{i},x_{j})=\text{exp}(\frac{-{\left\|x_{i}-x_{j}\right\|}^{2}}{2{\text{σ}}^{2}})$$

Therefore, there are two variables that need to be selected in the SVM model, which are the constant “*C*” and the width of the Gaussian RBF kernel “σ”. In this paper, IPSO is used to optimize the parameters of the SVM model.

### 3.2. Overview of Particle Swarm Optimization

The PSO is inspired by simulation of the behavior of birds flocking in nature [32,33]. It has won high reputation in numerical function optimization due to its precision and efficiency in the fields of stochastic optimization algorithms. It solves a problem by initializing a random population of candidate solutions (particles) and can search for the optimal solution through the search space. Each particle is characterized by a dynamic adjustable velocity, which originates from the momentum of a flying particle. Giving the PSO sufficient numbers of iterations, it is hoped to end up with a global optimal solution. The above-mentioned behavior of the *i*-th particles can be mathematically expressed as:

(9) $$V_{iD}^{t+1}=\text{ω}\times V_{iD}^{t}+c_{1}\times r_{1}\times ({pbest}_{iD}^{t}-X_{iD}^{t})+c_{2}\times r_{2}\times ({gbest}_{D}^{t}-X_{iD}^{t})$$

(10) $$X_{iD}^{t+1}=X_{iD}^{t}+V_{iD}^{t+1}$$

In the above formulas, ω denotes the inertia weight, $V_{iD}^{t}$ denotes its velocity vector of the *i*-th particle on dimension *D*; ${pbest}_{iD}^{t}$ denotes its previously best known position of the *i*-th particle on dimension *D* and ${gbest}_{D}^{t}$ denotes its best global position, *c*_1_ and *c*_2_ are social and personal learning parameters, *r*_1_ and *r*_2_ are random numbers in the range [0,1]; and $X_{iD}^{t}$ denotes its previous position of the *i*-th particle at time *t*.

### 3.3. Improved Particle Swarm Optimization

#### 3.3.1. The Basic Concept of the Cell Communication

Cell communication refers to a message through a medium to another cell and interacts with the corresponding receptors of target cells, and then through a series of physiological changes inside the cell. Indeed, cells cannot live in isolation, and survive by processing and receiving information from the external environment, whether that information suits the availability of nutrients and change in temperature. Cell to cell communication is essential for orchestration and coordination of cellular events in multi-cellular systems [34]. Intercellular communication has three main ways: gap junction, cell recognition, and chemical communication.

Gap junction: narrow water-filled channels that connect the cytoplasm of adjacent epithelial cells, as well as of some other cell types.

Cell recognition: mutual identification among cells, usually by particular complementary interaction among their separate membrane glycoproteins or surface molecules.

Chemical communication: cells secrete some chemicals (such as hormones) to outside the cell, as a signal molecule in target cells, adjusts its function, is specific and can be divided into four forms: (a) endocrine; (b) paracrine; (c) synaptic; (d) autocrine.

#### 3.3.2. IPSO Based on the Communication Mechanism of Cells

Although PSO has been applied to many kinds of practical problems, PSO still suffers to some degree of premature convergence and poor quality of solution. To overcome the shortcomings, we present IPSO by combining the cell communication mechanism. Based on the three main ways of cell communication, three evolutionary strategies are established. Specifically, the gap junction, the cell recognition, the chemical communication in the mode of cell communication can be replaced by evolutionary strategies, Equations (15)–(17) in the iterative and evolutionary process, respectively. IPSO applies modified update formula to maintain population diversity and enhances the convergence velocity and precision, which by incorporating the information of the global best solution into the search strategy improves the exploitation. In particular, the modified update formulas are determined as follows.

A solution of problem *P_i_* with *D* variables can be expressed as:

(11) $$\begin{array}{cc} P_{i}=[p_{i1},p_{i2},\cdot \cdot \cdot ,p_{iD}] & i=1,2,\cdot \cdot \cdot ,m \end{array}$$

**Figure 3:**
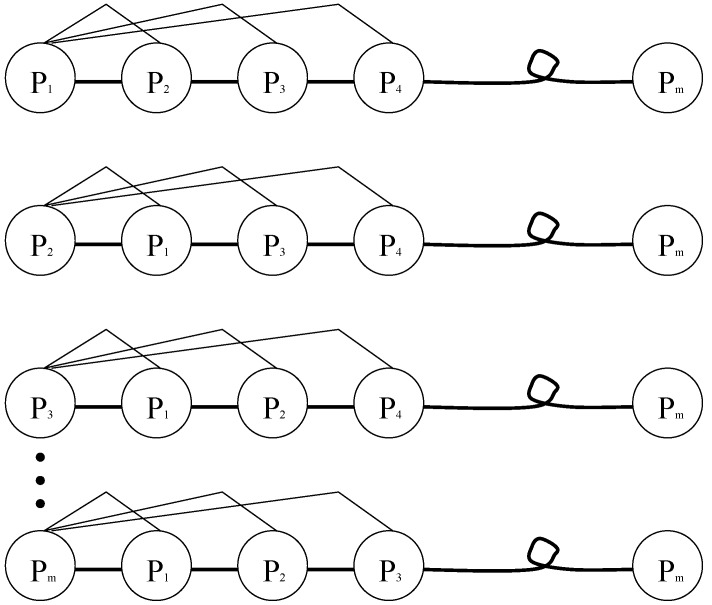
Schematic drawing to show distances with different solution.

An intuitive illustration of how the Euclidean distance is measured is given in Figure 3. As can be seen from Figure 3, the first line represents the Euclidean distance between the 1-th solution to all solutions, the second line represents the Euclidean distance between the 2-th solution to all solutions, and so forth. The Euclidean distance between solutions of problem can be defined as:

(12) $$d_{i,j}=\sqrt{\sum_{k=1}^{D}{\left(p_{ik}-p_{jk}\right)}^{2}}$$

(13) $$d_{ij}=\{\begin{array}{l} \begin{array}{cc} \begin{array}{c} 1 \end{array} & \begin{array}{cc} if & d_{i,j}\leq V_{\text{max}} \end{array} \end{array}\\ \begin{array}{cc} 0 & \begin{array}{c} otherwise \end{array} \end{array} \end{array}$$

The concentration of the particles can be expressed as:

(14) $$C_{k}=\frac{N_{k}}{N}$$

where *N_k_* denotes the total number of similar particles, and *N* denotes the total number of particles.

In this paper, two parameters of SVM are optimized, so, each particle’s position is checked using the following formula:

If (*C_k_* > 0.5):

(15) $$p_{i,1}=x_{b1}$$

where *p_i_*_1_ denotes the first value of the *i*-th particle; and *x_b_*_1_ denotes the first value of the best particle.

If ($f_{i}^{´}>f_{i}$):

(16) $$p_{i2}=x_{b2}$$

where $f_{i}$ denotes the fitness function value; $f_{i}^{´}$ denotes the updated fitness function value; *p_i_*_2_ denotes the second value of the *i*-th particle, and *x_b_*_2_ denotes the second value of the best particle.

The last particle’s position is checked using the following formula:

(17) $$P_{i}=x_{b}$$

where *P_i_* denotes the *i*-th particle; and *x_b_* denotes the best particle of the whole particles.

The values of the aforementioned parameters are usually prescribed to be selected as follows: ω = 0.8 and *c*_1_ = *c*_2_ = 2. Furthermore, ω denotes the particle’s search capability, and defines the balance between local search capabilities and global search capabilities. Once the velocity of the particle is calculated, its new position is updated according to Equation (10). The range of permissible velocity of the particle is usually restricted in order to eliminate fluctuations and obtain better exploration during the iteration process.

### 3.4. SVM Based on the IPSO

**Figure 4:**
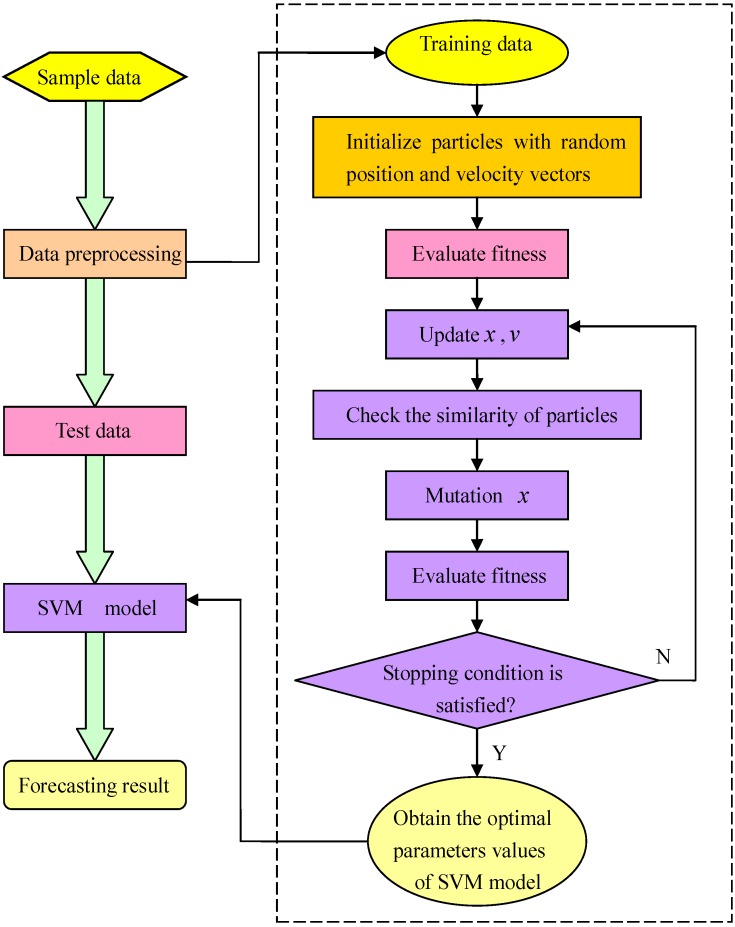
Diagram of the support vector machine and improved particle swarm optimization (SVM-IPSO) forecasting model.

The diagram of SVM-IPSO forecasting model is illustrated in Figure 4.

The details of IPSO for selecting parameters for the SVM model are as follows:

Step 1: Initialization parameters

The population size is 20, the maximum iteration number is 100, the initial particle swarm location is *X*, and the random flight velocity is *V* should first be determined. In the SVM-IPSO program, we set *X* = *rand*(1,2), where *rand*() is a random function in the range [0,1].

Step 2: Evolution starts

Set *gen* = 0, and give the random position and the flight velocity for food finding of an individual particle *i*. In the SVM-IPSO program, we use two variables [*x*_1_,*x*_2_] to denote the new position for food finding of an individual particle *i*.

Step 3: Preliminary calculations

Calculate the new position *X_i_* of an individual particle *i*, and then input *X_i_* into the SVM model for carbon fiber production forecasting; the parameters [*C*,σ] of the SVM model are replaced by [*X_i_*(1),*X_i_*(2)]. According to the carbon fiber production forecasting result, the *f_i_* can be calculated. The *f_i_* is represented by the root-mean-square error, as shown in Equation (18), which calculates the errors between the actual values and the forecasted values:

(18) $$f_{i}=\sqrt{\frac{1}{n}\sum_{i=1}^{n}{\left(y_{i}-{\hat{y}}_{i}\right)}^{2}}$$

where *n* is the number of training input data; *y_i_* is the *i*-th actual value; ${\hat{y}}_{i}$ denotes the *i*-th forecasting value.

Step 4: Offspring generation

The global best value is generated according to Equations (15)–(17) after updating the position value according to Equation (10). Then the global best value is inputted into the SVM model and the fitness function value calculated again. Set *gen* = *gen* + 1.

Step 5: Circulation stops

When *gen* is equal to the maximum iteration number, and the stop criterion is met, the optimal parameters of the SVM model are finally obtained. Otherwise, it is necessary to go back to Step 2.

## 4. Simulation and Discussion

### 4.1. The Preprocessing of Sample Data

In order to examine the performance of the new prediction model, we used the data organized from experiments in Table 1 [9]. The errors by the proposed method for the below mentioned experimental data, classified into four different types are given in Table 2, where to facilitate comparison the corresponding errors obtained by other methods on the same data are also listed.

**Table 1:** Experimental data of carbon fiber in the productive process.

No.	Viscosity Average Molecular Weight (10^4^)	Conversion Ratio (%)	Solid Content (%)	Spinning Jet Drawing Ratio (%)	Coagulating Bath Temperature (°C)	Total Drawing Ratio	Strength (CN/d)	Structure Parameter
1	8.9	94.5	20.8	−50.3	14	6.33	4.08	14.82
2	6.3	91.0	20.0	−59.7	15	5.89	3.23	12.63
3	11.6	92.0	20.4	−50.5	14	6.03	3.76	13.24
4	8.8	94.8	21.8	−63.4	13	6.65	4.17	17.24
5	7.0	81.8	17.9	−63.4	15	6.32	3.99	15.14
6	8.2	85.5	21.7	−59.5	15	5.49	4.58	16.61
7	7.2	89.8	19.5	−53.1	13	5.88	3.64	15.49
8	8.9	82.5	17.5	−56.8	19	6.38	4.07	17.57
9	8.0	83.4	18.6	−62.1	17	5.72	3.18	15.48
10	11.7	90.6	17.9	−53.8	16	6.47	3.22	12.10
11	11.5	82.8	18.7	−64.8	17	5.79	3.27	12.73
12	6.3	95.1	19.6	−54.9	16	6.37	4.36	17.18
13	10.4	98.6	20.2	−68.3	17	6.41	3.99	14.91
14	7.6	93.1	19.7	−55.4	16	5.88	3.38	17.07
15	8.5	84.6	22.3	−65.3	18	5.04	3.99	13.26
16	9.3	89.8	20.1	−53.8	16	5.66	3.30	15.31
17	11.7	79.4	22.7	−55.8	19	5.85	3.11	15.78
18	8.5	96.9	20.8	−51.8	14	5.54	4.70	12.19
19	11.9	96.4	22.7	−61.5	13	5.39	4.12	15.69
20	7.8	95.0	18.4	−63.7	13	6.64	4.86	14.17
21	10.2	82.7	21.1	−60.9	13	5.86	4.39	12.30
22	10.0	90.1	18.7	−58.5	15	6.78	4.17	14.94
23	9.2	77.5	21.0	−62.9	16	5.78	4.63	13.16
24	10.2	86.4	21.2	−63.0	15	6.54	4.76	12.74
25	10.0	83.9	17.4	−63.6	18	5.79	4.98	13.23
26	7.1	80.6	18.5	−62.7	17	6.62	3.00	12.88
27	6.8	80.9	18.3	−68.9	18	6.51	4.73	13.13
28	12.0	86.3	21.0	−54.2	19	5.75	4.23	12.26
29	7.0	79.1	22.1	−64.2	19	5.43	4.98	15.81
30	6.2	90.2	19.1	−54.7	14	6.58	4.06	13.69
31	9.4	87.4	21.7	−52.4	13	6.90	3.96	15.23
32	11.3	92.3	21.1	−62.1	17	5.66	4.60	16.17
33	10.0	92.4	17.0	−59.0	13	6.34	3.46	14.99
34	7.1	91.0	20.6	−59.2	16	5.88	4.00	15.21
35	8.2	77.7	19.3	−63.2	16	6.67	4.80	14.67
36	8.8	78.5	22.5	−65.4	19	6.54	4.15	12.74
37	11.9	84.0	17.0	−57.0	16	5.33	4.69	14.94
38	6.9	88.7	19.8	−63.2	15	6.72	4.48	17.12
39	11.1	91.4	19.5	−58.3	17	6.98	4.17	17.24
40	9.9	86.0	19.8	−66.8	18	6.03	3.49	13.62
41	8.3	95.0	21.6	−66.7	16	6.77	4.33	13.25
42	7.1	92.8	18.9	−55.1	15	6.18	3.17	15.39
43	8.6	98.3	21.7	−62.3	14	5.31	4.25	15.84
44	8.9	88.7	19.8	−61.6	17	5.40	4.32	14.50
45	6.7	84.2	17.2	−60.8	14	5.81	4.46	13.24
46	11.0	98.0	22.7	−74.6	12	6.89	3.90	12.82
47	9.5	92.2	20.3	−50.5	17	5.92	3.91	13.20
48	7.7	78.2	17.2	−63.4	19	6.17	3.99	15.19
49	9.5	79.3	18.1	−67.4	13	6.50	4.78	17.69
50	7.4	90.4	21.3	−55.3	18	6.65	4.96	12.49

Note: sample data sources come from reference [9].

**Table 2:** Comparison of errors among the proposed method, the GA-IPSO-RNN [9], the basic PSO-RNN [9], and the conventional RNN [9].

Algorithms		Conventional RNN	Basic PSO-RNN	GA-IPSO-RNN	Proposed method
MAE	1	1.1950	0.4818	0.4258	**0.3839**
	2	3.6827	2.0262	1.9833	**1.7821**
	Mean	2.4389	1.2540	1.2045	**1.0830**
MRE(%)	1	28.63	10.65	9.39	**8.71**
	2	27.96	14.61	14.01	**12.41**
	Mean	28.30	12.63	11.70	**10.56**
RMSE	1	1.4843	0.5841	0.5157	**0.4076**
	2	4.5364	2.2637	2.1177	**1.9649**
	Mean	3.0104	1.4239	1.3167	**1.1863**
TIC	1	0.1675	0.0690	0.0609	**0.0481**
	2	0.1452	0.0766	0.0727	**0.0679**
	Mean	0.1563	0.0728	0.0668	**0.0580**

Notes: 1: strength, 2: structure parameter.

In the SVM modeling, the preprocessing steps are implemented before attempting to calculate, to eliminate any bad data or outliers. These steps guarantee that the raw data retrieved from the database is completely suitable for modeling. In order to improve the accuracy of prediction and smooth the training procedure, all the sample data are normalized to fit them in the interval [0,1] using the following linear mapping formula:

(19) $$\begin{array}{cc} X^{´}=\left\{x_{i}^{´}\right\}=\frac{x_{i}-x_{\text{min}}}{x_{\text{max}}-x_{\text{min}}}, & i=1,2,3\cdot \cdot \cdot ,N \end{array}$$

where $x_{i}^{´}$ is the mapped value; $x_{i}$ is the initial value from the experimental data; *N* is the total number of input data; *x*_max_ and *x*_min_ denote the maximum and minimum values of initial data, respectively.

In Table 1, the values for the first six columns indicate different parameters, and the values for the last two columns indicate different property indices. These detailed parameters can synthetically represent the whole manufacturing process. Meanwhile, the two kinds of property indices can fully represent the performance of the carbon fiber. In the predictive process, we obtained training sets from the first 45 rows of samples and test sets from the last five rows of samples. Also, to examine the performance of the several algorithms, the mean absolute error, root mean square error, mean relative error and Theil’s inequality coefficient were defined according to the following formulations, respectively:

Mean absolute error:

(20) $$MAE=\frac{1}{n}\sum_{i=1}^{n}\left|y_{i}-{\hat{y}}_{i}\right|$$

Root mean square error:

(21) $$RMSE=\sqrt{\frac{1}{n}\sum_{i=1}^{n}{\left(y_{i}-{\hat{y}}_{i}\right)}^{2}}$$

Mean relative error:

(22) $$MRE=\frac{1}{n}\sum_{i=1}^{n}|\frac{y_{i}-{\hat{y}}_{i}}{y_{i}}|$$

Theil’s inequality coefficient:

(23) $$TIC=\frac{\sqrt{\sum_{i=1}^{n}{\left(y_{i}-{\hat{y}}_{i}\right)}^{2}}}{\sqrt{\sum_{i=1}^{n}{\left(y_{i}\right)}^{2}}+\sqrt{\sum_{i=1}^{n}{\left({\hat{y}}_{i}\right)}^{2}}}$$

where *y_i_* is the true value; ${\hat{y}}_{i}$ is the predicted value.

### 4.2. The Selection of Comparative Models

As mentioned above, the technological process for carbon fiber is a nonlinear system, it involves many process parameters that must be carefully optimized and adjusted. Most of the previous work was focused on analyzing the properties of carbon fibers by means of physical or chemical instruments. However it is a kind of method which wastes time and energy. Thus, we should build the predictive model for the productive process of carbon fiber. In this way, we can effectively predict the properties of carbon fiber in real-time, and thus we can find out inferior carbon fiber with poor quality in advance, timely adjust the process parameters, and avoid wasting raw materials. However, on the contrary, the prediction of productive parameters by the properties of the carbon fiber is a backward reasoning process. Therefore, it is a better idea to analyze all antecedent production schemes through computer simulation. This will avoid partial production schemes created by operational staff who rely on their personal experience and interests.

### 4.3. Forward Prediction

**Figure 5:**
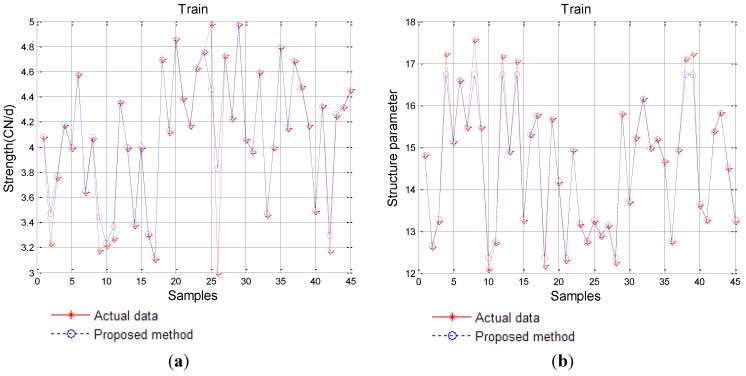
Training results of the proposed SVM-IPSO model. (**a**) Strength; (**b**) Structure parameter.

Carbon fiber production is a positive process, and the predictable performance of carbon fiber by productive parameters is a forward prediction, too. Thus, in forward prediction, we can take the parameters of carbon fiber production as input and the corresponding performance of carbon fiber as output. By employing IPSO to optimize the parameters of SVM, we gain the best parameters for SVM. The training effectiveness of the proposed method is shown in Figure 5.

Comparing performance of the conventional RNN, the basic PSO-RNN, the GA-IPSO-RNN with the SVM-IPSO is carried out in terms of (a) mean absolute error; (b) root mean square error; (c) mean relative error and (d) Theil’s inequality coefficient result. The detailed results are listed in Table 2.

From Table 2, we can see that on the basis of *MAE*, the proposed SVM-IPSO achieves roughly a decrease of 55.59%, 13.64% and 10.09% compared to the other three methods, respectively. Similar results can be obtained from the other errors such as *MRE* with a decrease of 62.69%, 16.39% and 9.74%, *RMSE* with a decrease of 60.59%, 16.69% and 9.90%, and *TIC* with a decrease of 62.89%, 20.33% and 13.17%. Similar results are obtained on the basis of strength and structure parameters.

### 4.4. Backward Prediction

**Figure 6:**
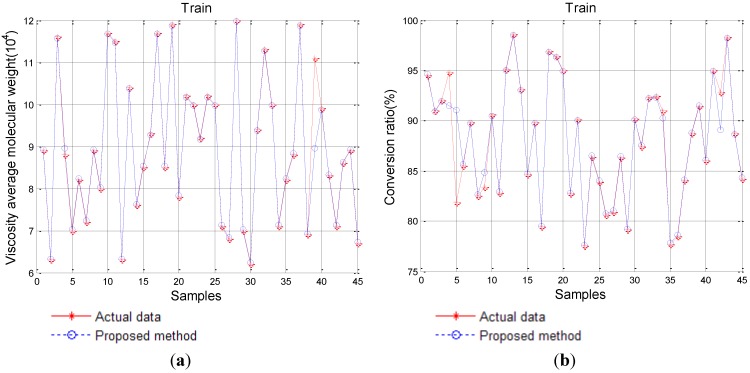

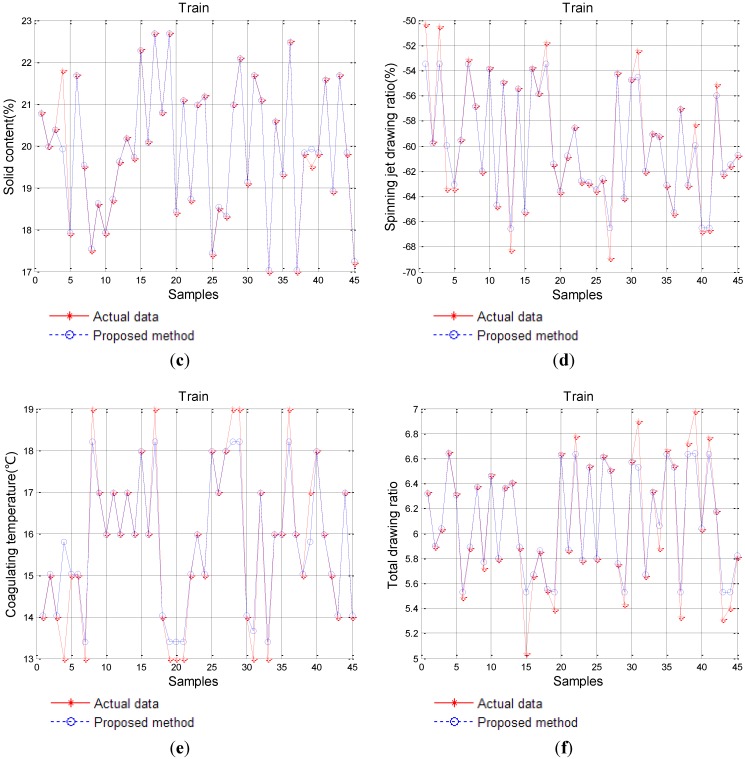
Training results of the proposed SVM-IPSO model. (**a**) Viscosity average molecular weight; (**b**) Conversion ratio; (**c**) Solid content; (**d**) Spinning jet drawing ratio; (**e**) Coagulating temperature; (**f**) Total drawing ratio.

The productive parameters of carbon fiber forecast by the performance of carbon fiber is a reverse process of forward prediction, so the predictive process can be considered inverse inference. In the backward prediction, we take the performance of carbon fiber as input and the productive parameters as output, which can be considered as a design scheme for a novel style carbon fiber. In order to achieve the above goals, by employing IPSO to optimze the parameters of SVM, we gain the best parameters for SVM. The training effectiveness of the proposed method is shown in Figure 6.

We compared the effectiveness of SVM-IPSO with that of conventional RNN, the basic PSO-RNN, and the GA-IPSO-RNN optimal schemes. The detailed results are listed in Table 3. In Table 3, (1) is the viscosity average molecular weight; (2) is the conversion ratio; (3) is the solid content; (4) is the spinning jet drawing ratio; (5) is the coagulating bath temperature; (6) is the total drawing ratio.

From Table 3, we can see that on the basis of *MAE*, the proposed SVM-IPSO achieves roughly a decrease of 98.69%, 19.48% and 6.68% compared to the other three methods, respectively. Similar results can be obtained from the other errors such as *MRE* with a decrease of 98.72%, 25.78% and 7.51%, *RMSE* with a decrease of 99.22%, 18.70% and 7.60%, and *TIC* with a decrease of 93.02%, 23.26% and 11.48%. Similar results are obtained on the basis of viscosity average molecular weight, conversion ratio, solid content, spinning jet drawing ratio, and coagulating bath temperature, respectively.

**Table 3:** Comparison of errors among the proposed methods, the GA-IPSO-RNN [9], the basic PSO-RNN [9], and the conventional RNN [9].

Algorithms	MAE	MRE (%)	RMSE	TIC
Conventional RNN				
1	173.1400	1876.58	341.7052	0.9612
2	1062.8000	1310.66	2004.3000	0.9391
3	151.6600	816.09	285.3062	0.9051
4	171.6800	264.56	285.1956	0.7416
5	62.6800	443.07	103.5493	0.8054
6	34.8800	533.97	63.3625	0.9510
Mean	276.1367	874.16	513.9031	0.8839
Basic PSO-RNN				
1	1.9652	22.73	2.2049	0.1138
2	9.7604	11.30	10.3417	0.0590
3	2.5932	13.19	2.7487	0.0690
4	8.9433	15.27	10.2110	0.0806
5	3.2098	20.66	3.2757	0.1054
6	0.4920	7.38	0.6731	0.0544
Mean	4.4940	15.09	4.9092	0.0804
GA-IPSO-RNN				
1	1.3996	14.37	1.7597	0.1026
2	8.2660	9.61	9.1148	0.0518
3	2.2693	11.04	2.5107	0.0647
4	8.0976	13.47	9.1473	0.0733
5	2.9085	19.20	2.9776	0.0944
6	**0.3260**	**4.94**	**0.4054**	**0.0317**
Mean	3.8778	12.11	4.3192	0.0697
Proposed method				
1	**1.0585**	**11.80**	**1.2290**	**0.0693**
2	**7.7362**	**9.03**	**8.4285**	**0.0479**
3	**1.7943**	**9.17**	**1.9964**	**0.0500**
4	**7.9765**	**12.74**	**8.8876**	**0.0724**
5	**2.7471**	**18.42**	**2.9189**	**0.0920**
6	0.3994	6.02	0.4855	0.0387
Mean	**3.6187**	**11.20**	**3.9910**	**0.0617**

## 5. Conclusions

The non-linear relationship of the productive parameters for carbon fiber with its performance makes bi-directional prediction very complicated. Thus, how to improve the bi-directional prediction accuracy is worthy of study. In this paper, we present the SVM-IPSO hybrid algorithm to bi-directionally forecast the productive process for carbon fiber, which can be considered as predicting the performance of carbon fiber and designing a method for new carbon fiber production. By the proposed model, we can online forecast the performance of the carbon fiber, and adjust the productive parameters in time, while reducing time and energy. In addition, this model can provide a means to determine the productive parameters for production to meet the performance requirements of different fibers.
